# Association between elevated serum amyloid a levels and clinical outcomes in intracerebral haemorrhage: a retrospective study

**DOI:** 10.3389/fneur.2026.1806993

**Published:** 2026-07-09

**Authors:** Jixin He, Tongzhang Xu, Rong Wu, Ying Hu, Dan Zhao, Wenbo Zhang, Fangfang Wang, Jie Cao, Min Jiang, Xiaoping Yin, Moxin Wu

**Affiliations:** 1 Department of Medical Laboratory, Affiliated Hospital of Jiujiang University, Jiujiang, China; 2 Jiujiang Clinical Precision Medicine Research Center, Jiujiang, China; 3 School of Economics and Management, Jiangxi Polytechnic University, Jiujiang, China; 4 JXHC Key Laboratory of Screening and Diagnosis of Maternal and Child Genetic Disease, Jiujiang Maternal and Child Healthcare Hospital, Jiujiang, China; 5 Department of Pathology, Lushan Rehabilitation and Recuperation Center, Jiujiang, China; 6 Department of Neurology, Affiliated Hospital of Jiujiang University, Jiujiang, China

**Keywords:** dynamically monitored, haematoma expansion, intracerebral haemorrhage, secondary brain injury, serum SAA levels

## Abstract

**Objective:**

Following an intracerebral haemorrhage (ICH), secondary brain injury (SBI) occurs, triggering an inflammatory response. Serum amyloid A (SAA) levels, an acute-phase response protein, increase during acute inflammation and tissue damage. While SAA is associated with ICH prognosis, systematic studies investigating its dynamic longitudinal progression and its relationship with bleeding severity remain limited.

**Materials and methods:**

In this single-centre, real-world cohort study, we measured SAA levels in 554 ICH patients and 119 healthy controls. Neurological deficit was quantified based on haematoma expansion (HE), the National Institutes of Health Stroke Scale (NIHSS) score, and the Glasgow Coma Scale (GCS) score after ICH. SAA levels were measured within 24 h of admission and on days 3 and 7 after ICH onset in this longitudinal study. Univariate and multivariate logistic regression analyses were performed to determine the independent association between SAA levels and NIHSS and GCS scores. Adjusted logistic regression and receiver operating characteristic (ROC) curves were used to assess the association between HE and SAA levels. Additionally, the role of SAA levels in SBI was evaluated by monitoring their changes within 24 h of admission and on days 3 and 7 after ICH.

**Results:**

SAA levels were significantly higher in patients with ICH than in healthy controls (3.1 vs. 2.4 mg/L, *p* < 0.001). SAA levels were positively correlated with disease severity (NIHSS: *r* = 0.106, 95% CI: 0.011–0.200, *p* = 0.029; GCS: *r* = −0.128, 95% CI: −0.213 to −0.041, *p* = 0.003) and were independently associated with a poor prognosis. The AUC for the diagnostic performance of SAA levels in HE was 0.694 (95% CI: 0.630–0.758). Furthermore, SAA levels were significantly elevated on days 3 and 7 compared with the levels at admission (*p* < 0.001).

**Conclusion:**

Patients with ICH exhibit elevated SAA levels that are associated with prognosis. Dynamic monitoring of these levels indicates that day 3 may be a critical time point for observing changes in SAA levels.

## 1 Introduction

Spontaneous intracerebral haemorrhage (ICH) is a sudden cerebrovascular condition that leads to high disability rates and has a mortality rate of 30–40% (1). The global incidence of ICH is 40.83 per 100,000 people, accounting for 28.8% of all strokes (2). After discharge, survivors often experience multiple irreversible functional injuries and long-term complications (3). Although managing blood pressure and performing haematoma evacuation can reduce ICH-related disability and mortality, the overall prognosis is still poor (2). Following ICH, preventing secondary brain injury (SBI) becomes the primary focus of treatment for patients. SBI typically manifests as microcirculation dysfunction, blood–brain barrier (BBB) disruption, neuroinflammation, oxidative stress cascades, brain oedema, and neuronal cell death (4). After haemorrhage occurs in the brain, the immune cells surrounding the haematoma are rapidly activated, triggering an inflammatory cascade and exacerbating neurological dysfunction.

Neuroinflammatory responses are characterised by the activation of neuroinflammatory cells and other factors (5). These neuroinflammatory cells include microglia, astrocytes, and infiltrating neutrophils and lymphocytes (6); neuroinflammatory factors include interleukin-1β (IL-1β) and tumour necrosis factor-*α* (TNF-α). In addition, levels of acute-phase proteins such as C-reactive protein (CRP) and serum amyloid A (SAA) may be elevated during neuroinflammation. These markers are frequently used to predict the prognosis of various neuroinflammatory diseases. SAA is an acute-phase reactant and serves as a key rapid-response marker of inflammation (7). It can induce a cascade of inflammatory mediators characterised by systemic, multiorgan, and local vascular effects. In healthy individuals, SAA concentrations are <10 mg/L. Following an infection or tissue injury, these levels can increase within 3–6 h, potentially reaching 10–1,000 times the normal range. The degradation products of SAA can accumulate in organs, leading to the formation of amyloid fibres. Clinical studies have shown that elevated SAA levels in patients with acute ischaemic stroke (AIS) at admission can serve as a prognostic indicator of poor functional outcomes following thrombolytic therapy (8). Specifically, elevated SAA levels at admission predict poor outcomes and cognitive impairment 3 months after receiving intravenous thrombolysis (8, 9). In cases of neonatal hypoxic–ischaemic encephalopathy, elevated SAA levels are associated with the severity of brain injury and increased mortality (10). Three months after aneurysmal subarachnoid haemorrhage, high levels of SAA are also associated with poor prognosis (11). Similarly, in patients with acute primary basal ganglia haemorrhage, elevated SAA levels are associated with mortality and poor prognosis (12). However, systematic investigations into the dynamic longitudinal progression of SAA levels and their association with haemorrhage severity remain limited.

This retrospective study investigated the relationship between SAA levels and clinical outcomes in ICH patients. By monitoring its dynamic levels, we aim to gain a better understanding of disease progression and determine whether SAA may serve as a potential predictor of clinical prognosis in ICH patients.

## 2 Materials and methods

### 2.1 Study design

This real-world, retrospective cohort study was conducted following the STROBE statement (13). This study included patients who were diagnosed with their first acute spontaneous ICH via head computed tomography (CT) scans and who were hospitalised at the Affiliated Hospital of Jiujiang University (Jiujiang, China) from January 2021 to December 2024. All patients were hospitalised within 24 h of their ICH diagnosis and received non-surgical treatment for their haematomas. The exclusion criteria were as follows: (1) age <18, (2) being pregnant, (3) having a history of cerebral infarction, (4) having secondary cerebral haemorrhage, (5) having an indication for surgery, and (6) having missing data (e.g., SAA or glucose). Concurrently, healthy controls were selected to match the ICH group in terms of age and sex. The control group underwent routine laboratory examinations and had no history of ICH. ICH patients in the SAA-monitoring group had complete SAA measurements taken within 24 h on days 3 and 7. All patients were admitted within 24 h of experiencing ICH, had no history of ICH or active infection, and had completed SAA sample collection at all time points. This study was approved by the Institutional Review Committee of the Affiliated Hospital of Jiujiang University (Approval No. IRB2025-JJU-04-21). Patient informed consent is not required for retrospective studies according to national legislation and institutional requirements.

### 2.2 Data collection

Peripheral venous blood samples were collected from individuals in the ICH and control groups within 24 h of admission. Demographic data (age and sex), vascular risk factors (hypertension and diabetes), lifestyle factors (smoking and alcohol consumption), and laboratory parameters (white blood cell count and blood glucose, serum potassium, and SAA levels) were recorded for both groups.

### 2.3 Detection of SAA levels

Peripheral venous blood was collected from patients with ICH within 24 h of admission and on days 3 and 7 after ICH. For the measurement of SAA levels, blood samples were centrifuged at 3000 × g for 5 min, and the serum was separated in a clinical laboratory for analysis. SAA concentrations were quantitatively determined according to the manufacturer’s instructions for the Beckman Coulter reagent package, using a Beckman Coulter AU5800 clinical chemical analyser (Brea, Beckman Coulter, California, United States), and the measurement system remained unchanged throughout the study period. The measurements were performed by the same technician, who was blinded to the clinical data.

### 2.4 Clinical outcome evaluation

On admission, disease severity was assessed by a trained neurologist using the NIHSS and GCS scores (14, 15). For the assessment of the haematoma volume, semiautomated CT volumetric measurements were performed using ITK-SNAP software (University of Pennsylvania, Philadelphia, United States; www.itksnap.org). HE was defined as haematoma growth of >33% and/or >6 mL within 24 h compared with baseline. In accordance with the neurocritical care guidelines, coma was defined as a GCS score of ≤8 at discharge. Prognosis was determined based on the occurrence of in-hospital death and coma at discharge.

### 2.5 Statistical analysis

Statistical analysis was performed using GraphPad Prism version 10.1.2 (GraphPad Software, La Jolla, CA, United States). The normality of the quantitative data was assessed using the Kolmogorov–Smirnov test. Depending on whether the data were normally distributed, either the independent t-test (for normally distributed data) or the Mann–Whitney U-test (for non-normally distributed data) was used. Normally distributed data were reported as means ± standard deviations, and non-normally distributed data were summarised as medians with upper and lower quartiles. Comparisons between two groups were performed using unpaired *t*-tests or paired *t*-tests. The Kruskal–Wallis H test was performed to compare multiple datasets. Categorical variables were expressed as the number of cases (percentages), and comparisons between two groups were performed using the *χ2* test. The median HE, NIHSS, and GCS scores were defined as critical values. Spearman’s correlation analysis was used to analyse the associations between SAA levels and clinical parameters (HE, NIHSS, and GCS scores). Multivariate logistic regression was used to analyse the correlation between SAA levels and clinical outcomes. A log transformation [log (SAA + 1)] was applied to SAA in the regression analysis. The predictive performance of SAA was evaluated by plotting the ROC curve (16). A multivariable logistic regression model was used to assess the effect of SAA levels on HE. To ensure the robustness of the findings, multiple imputation was performed to assess the sensitivity to missing data. The variance inflation factor (VIF) was calculated to determine multicollinearity. To reduce potential selection bias, the baseline characteristics were compared between the SAA-monitoring and non-monitoring groups. Missing data were not imputed. A two-tailed *p*-value of <0.05 was considered statistically significant.

## 3 Results

### 3.1 Patient selection and characteristics

**Figure 1:**
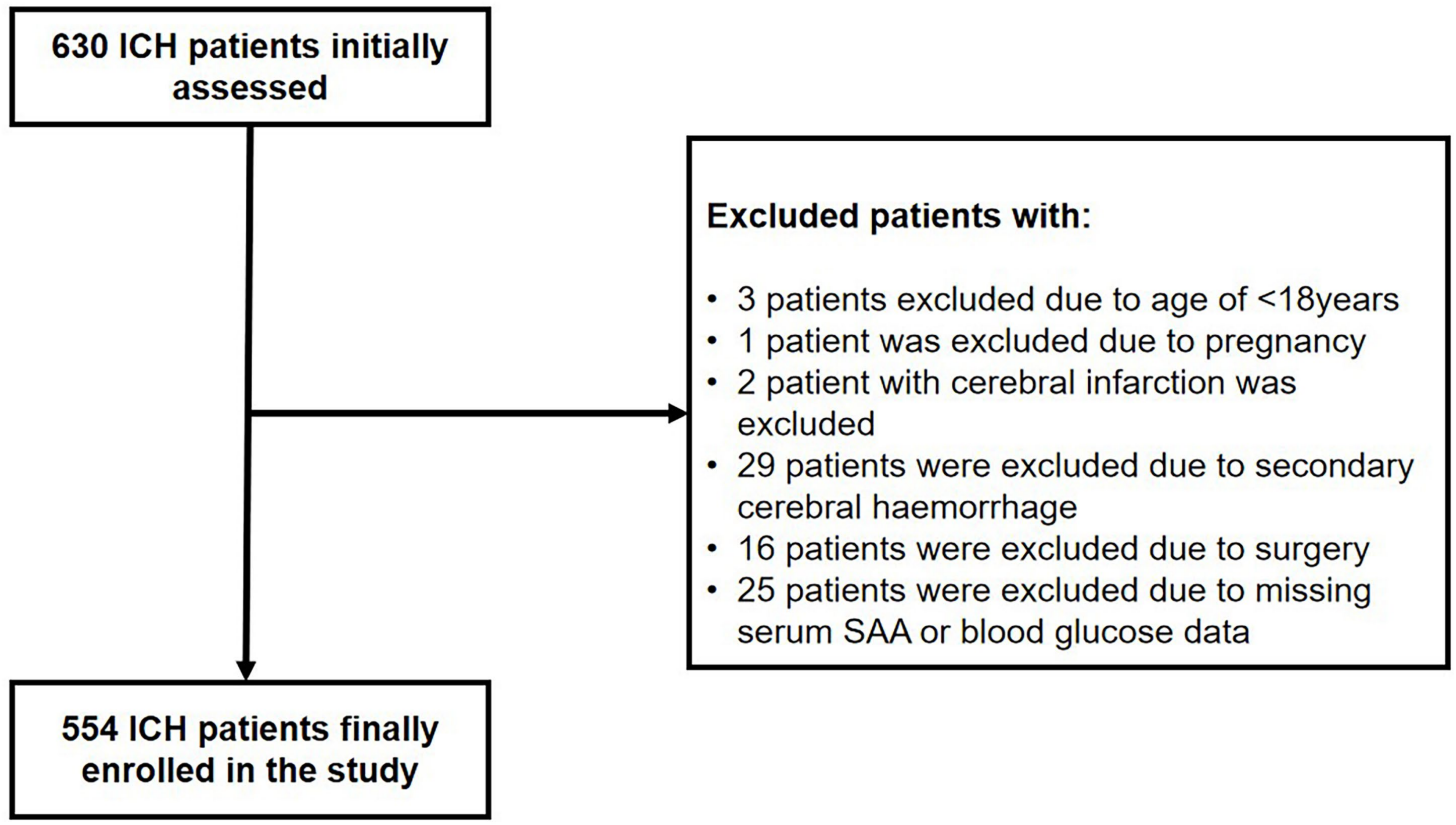
Flowchart for the screening of eligible patients with acute spontaneous ICH in this study. Initially, 630 ICH patients were assessed; thereafter, 76 patients were excluded. Ultimately, 554 ICH patients were enrolled. ICH, intracerebral haemorrhage.

In this study, 630 patients with spontaneous ICH who were admitted to the hospital within 24 h of symptom onset were initially included. However, 76 patients were excluded based on the exclusion criteria, as shown in Figure 1. Finally, 554 patients with ICH (358 men and 196 women) were enrolled in this study. Additionally, 119 healthy controls (77 men and 42 women) were recruited. The mean ages of patients with ICH and healthy controls were 63.64 ± 13.29 years and 60.83 ± 13.36 years, respectively. No significant differences were noted between ICH patients and healthy controls in terms of sex (*p* = 0.986), age (*p* = 0.155), diabetes mellitus (*p* = 0.731), current smoking (*p* = 0.991), and alcohol consumption (*p* = 0.771). As expected, patients with ICH had a higher prevalence of hypertension (*p* < 0.001) and significantly elevated serum glucose levels (8.64 ± 3.13 vs. 7.03 ± 3.97, *p* < 0.001). In addition, patients with ICH had elevated white blood cell counts (9.50 ± 4.20 vs. 6.48 ± 2.34, *p* < 0.001) and decreased serum potassium concentrations (3.80 ± 0.49 vs. 4.35 ± 0.37, *p* < 0.001). SAA data for both the ICH group (K-S = 0.356; *p* < 0.001) and the healthy control group (K-S = 0.096; *p* = 0.009) showed that neither group followed a normal distribution (Supplementary Figure S1), and the SAA concentrations in ICH patients were significantly higher than those in the control group [3.1 (1.3, 9.1) mg/L vs. 2.1 (0.9, 3.5) mg/L, *p* < 0.001]. Table 1 shows the demographic and clinical characteristics of ICH patients.

**Table 1:** Demographic data and vascular risk factors for ICH patients and controls.

Characteristic	Control (*n* = 119)	ICH (*n* = 554)	*p*-value
Male patients, n (%)	77(64.7%)	358(64.6%)	0.986
Age, years (mean ± SD)	60.83 ± 13.36	63.64 ± 13.29	0.155
Hypertension, *n* (%)	37(31.1%)	346(62.5%)	< 0.001***
Diabetes mellitus, *n* (%)	11(9.2%)	57(10.3%)	0.731
Current smoking, *n* (%)	24(20.2%)	112(20.2%)	0.991
Alcohol consumption, *n* (%)	17(14.3%)	85(15.3%)	0.771
Serum glucose level (mmol/L)	6.21 ± 2.63	8.64 ± 3.13	< 0.001***
Serum potassium level (mmol/L)	4.33 ± 0.43	3.80 ± 0.49	< 0.001***
Blood leucocyte count (×10^9^/L)	6.30 ± 2.09	9.50 ± 4.20	< 0.001***
Serum SAA levels (mg/L)	2.1 [0.9, 3.5]	3.1 [1.3, 9.1]	< 0.001***

Age is reported as the mean ± standard deviation, and qualitative data are presented as counts (proportions). Intergroup comparisons of various variables were conducted using the *χ2* test for qualitative data and the Mann–Whitney U-test for quantitative data. Variables with non-normal distributions are characterised by the median (interquartile range). An asterisk indicates statistical significance (*p < 0.05). ICH, intracerebral haemorrhage.

### 3.2 Elevated SAA levels and their correlation with clinical outcomes

**Figure 2:**
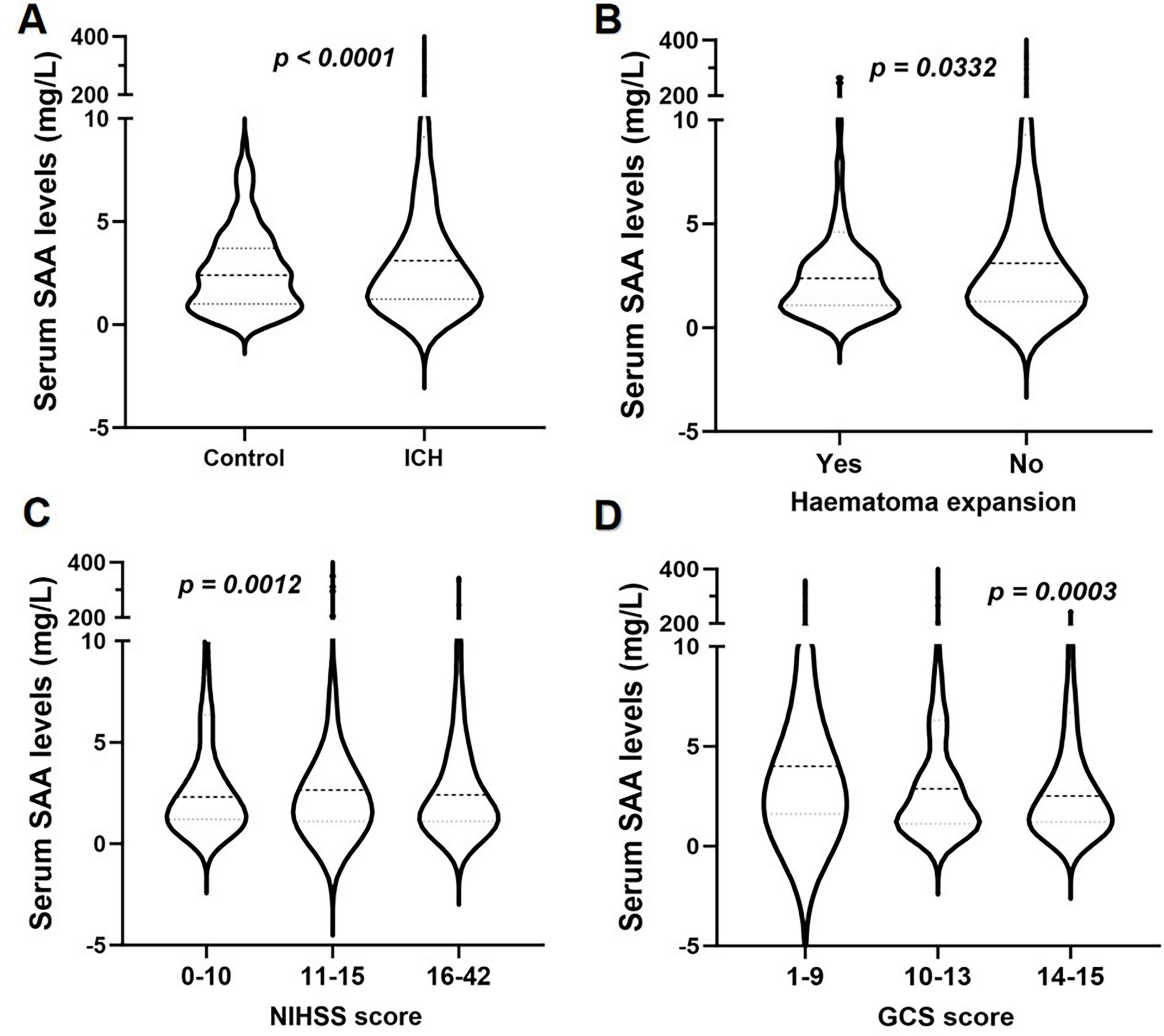
Serum SAA levels in patients with ICH were related to ICH severity. Serum SAA levels are reported as medians (upper and lower quartiles). **(A)** Serum SAA levels in patients with ICH and healthy controls. Relationship between serum SAA levels and **(B)** haematoma expansion, **(C)** NIHSS score, and **(D)** GCS score in patients with acute ICH. ICH, intracerebral haemorrhage; NIHSS, National Institutes of Health Stroke Scale; GCS, Glasgow Coma Scale.

The SAA concentrations were higher in ICH patients [3.1 (1.3, 9.1) mg/L] than in healthy controls [2.4 (1.0, 3.7) mg/L; *p* < 0.001] (Figure 2A). To clarify the relationship between SAA levels and the severity of haemorrhage, we analysed the HE levels, NIHSS scores, and GCS scores of patients with ICH. Interestingly, SAA levels were lower in ICH patients with HE than in those without HE (*p* = 0.033) (Figure 2B). Similarly, SAA levels were significantly increased in patients with higher NIHSS scores (*p* = 0.001) and lower GCS scores (*p* < 0.001) (Figures 2C, D).

**Figure 3:**
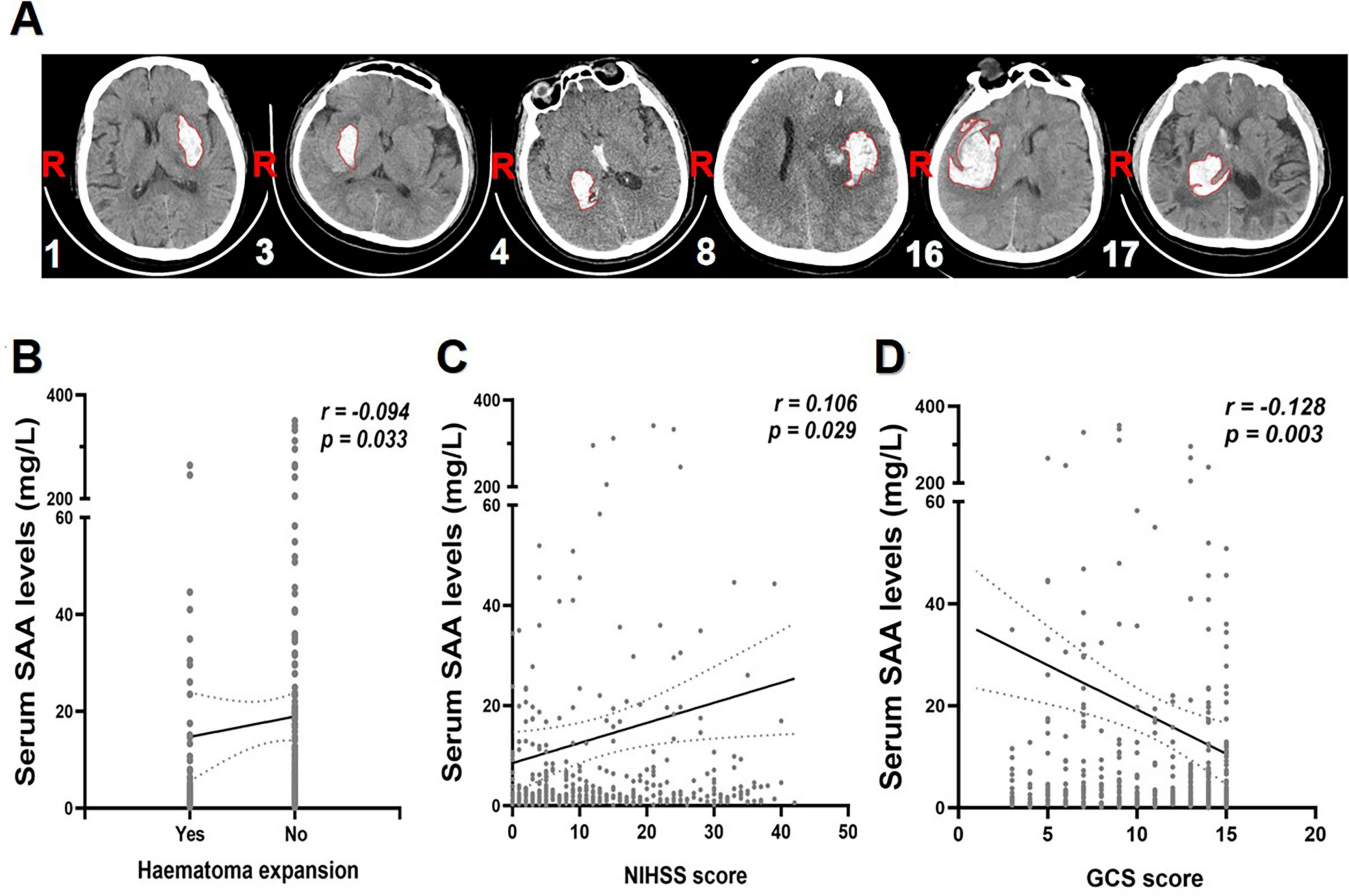
Serum SAA levels were positively correlated with the clinical prognosis of patients with ICH. **(A)** CT images of patients with ICH for analysis; the corresponding numbers of patients are listed in the lower left corner of each image. **(B)** Correlations between haematoma expansion **(C)** and the NIHSS and **(D)** GCS scores. Spearman’s correlation coefficients were used for the correlation analysis. ICH, intracerebral haemorrhage; NIHSS, National Institutes of Health Stroke Scale; GCS, Glasgow Coma Scale.

**Figure 4:**
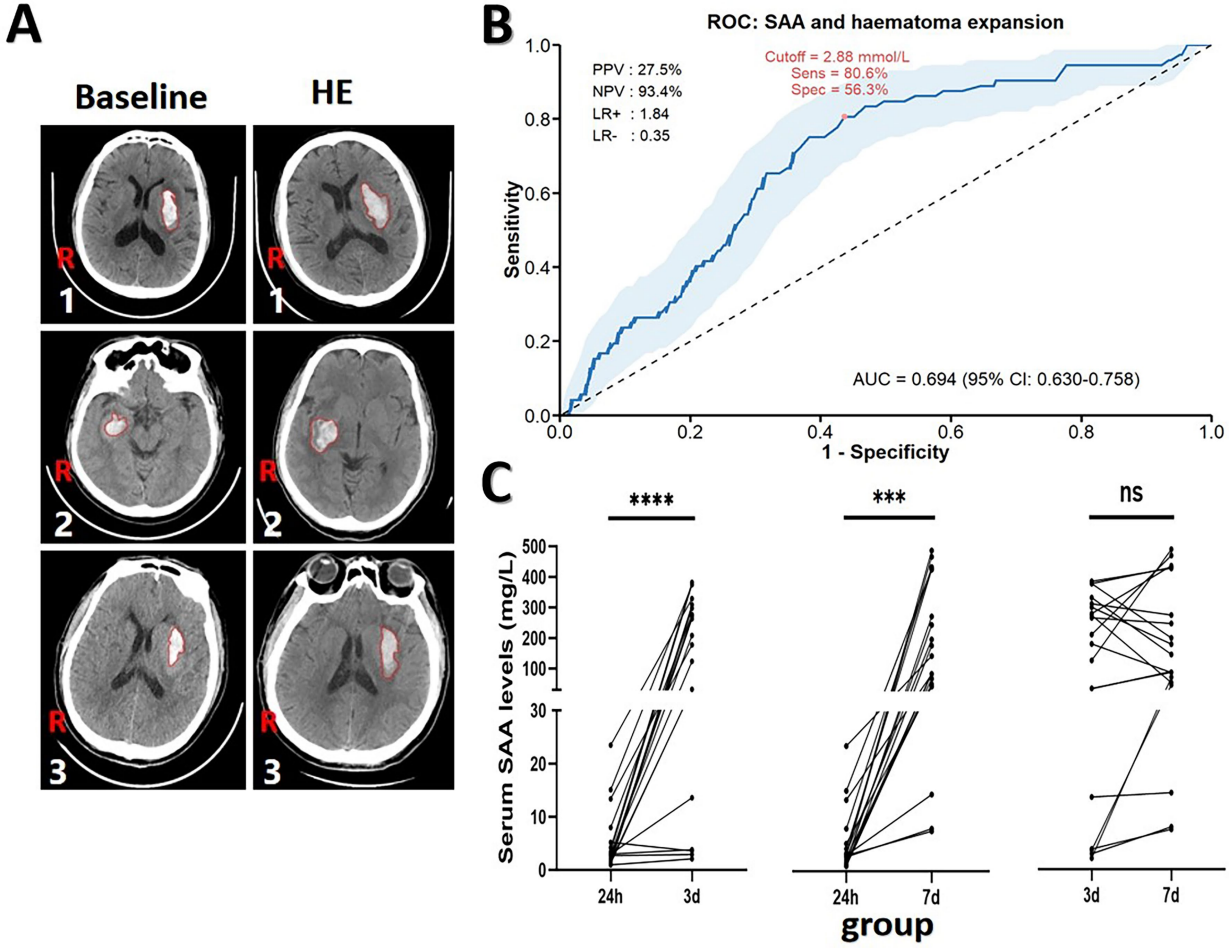
Elevated serum SAA levels are associated with the occurrence of secondary brain injury after ICH. ICH patients were monitored dynamically within 24 h after admission and on days 3 and 7. **(A)** Baseline and HE CT images. **(B)** ROC curve of serum SAA levels for HE. **(C)** Paired *t*-tests were used to analyse the serum SAA levels of ICH patients within 24 h of admission and on days 3 and 7. ICH, intracerebral haemorrhage; HE, haematoma expansion; ROC, receiver operating characteristic; AUC, area under the curve.

Spearman’s correlation coefficient was used to determine the correlations between SAA levels and HE, NIHSS, and GCS scores. Figure 3A shows the CT imaging diagnoses of the patients included in the analysis. In ICH patients, SAA was positively correlated with the NIHSS score (*r* = 0.106; 95% CI: 0.011–0.200; *p* = 0.029). Additionally, SAA was negatively correlated with HE (*r* = −0.094, 95% CI: 0.005–0.182, *p* = 0.033) and GCS scores (*r* = −0.128; 95% CI: −0.213 to −0.041; *p* = 0.003) (Figures 3B–D). In 425 patients, multivariate logistic regression analysis showed that higher SAA levels were independently associated with higher NIHSS scores [odds ratio (OR) = 1.01; 95% CI: 1.00–1.02; *p* = 0.021] and lower GCS scores (OR = 0.99; 95% CI: 0.99–1.00; *p* = 0.006) in ICH patients (Table 2). To assess the predictive performance of SAA levels, the area under the curve (AUC) derived from ROC curves was calculated. The AUC for SAA levels and HE was 0.694 (95% CI: 0.630–0.758). The cut-off value was 2.88 mmol/L, with a sensitivity of 80.6% and a specificity of 56.3%. The positive predictive value (PPV) was 27.5%, the negative predictive value (NPV) was 93.4%, the positive likelihood ratio (LR+) was 1.84, and the negative likelihood ratio (LR-) was 0.35 (Figure 4B). In the validation analysis (Supplementary Figure S2), the sensitivity analysis supported the reliability of the results, and all the VIF values were <2, indicating no significant multicollinearity. Overall, the results indicated that elevated clinical SAA levels were significantly and independently associated with haemorrhage severity.

**Table 2:** Univariate and multivariate logistic risk analyses in patients with ICH.

Clinical characteristics	Univariate analysis						Multivariate analysis					
	Haematoma expansion		NIHSS score		GCS score		Haematoma expansion		NIHSS score		GCS score	
	OR (95% Cl)	*p*-value	OR (95% Cl)	*p*-value	OR (95% Cl)	*p*-value	OR (95% Cl)	*p*-value	OR (95% Cl)	*p*-value	OR (95% Cl)	*p*-value
Age	0.99 (0.97–1.01)	0.302	0.99 (0.98–1.01)	0.581	1.01 (0.99–1.02)	0.267	0.99 (0.97–1.01)	0.373	0.99 (0.98–1.01)	0.499	1.01 (0.99–1.03)	0.421
Gender	0.84 (0.48–1.44)	0.542	0.88 (0.58–1.34)	0.561	0.76 (0.50–1.15)	0.189	0.61 (0.32–1.13)	0.121	1.06 (0.65–1.72)	0.812	0.90 (0.56–1.47)	0.680
Hypertension	2.24 (1.34–3.75)	0.002**	0.69 (0.46–1.06)	0.086	1.33 (0.87–2.05)	0.190	2.11 (1.22–3.66)	0.008**	0.71 (0.45–1.10)	0.126	1.28 (0.81–2.05)	0.289
Diabetes mellitus	2.26 (0.87–7.69)	0.133	0.59 (0.28–1.16)	0.144	1.91 (1.03–3.44)	0.034*	1.89 (0.58–7.85)	0.330	1.05 (0.44–2.41)	0.911	1.17 (0.53–2.48)	0.693
Current smoking	1.64 (0.85–3.43)	0.158	0.70 (0.43–1.14)	0.151	0.62 (0.35–1.04)	0.079	2.57 (1.10–6.51)	0.036*	0.87 (0.46–1.67)	0.680	0.66 (0.32–1.30)	0.237
Alcohol consumption	1.12 (0.58–2.37)	0.743	1.53 (0.89–2.60)	0.117	0.73 (0.40–1.29)	0.301	0.91 (0.38–2.27)	0.830	1.31 (0.66–2.61)	0.435	1.15 (0.54–2.38)	0.707
Serum glucose level	1.02 (0.94–1.14)	0.623	0.80 (0.72–0.88)	< 0.001***	1.21 (1.14–1.29)	< 0.001***	0.99 (0.88–1.12)	0.885	0.83 (0.74–0.92)	< 0.001***	1.14 (1.05–1.24)	0.002**
Serum potassium level	0.68 (0.42–1.13)	0.129	1.21 (0.79–1.85)	0.375	0.68 (0.43–1.04)	0.080	0.75 (0.43–1.35)	0.324	1.14 (0.71–1.83)	0.587	0.79 (0.49–1.25)	0.323
Blood leucocyte count	0.97 (0.91–1.04)	0.405	0.90 (0.85–0.95)	< 0.001***	1.16 (1.11–1.22)	< 0.001***	0.99 (0.92–1.08)	0.896	0.93 (0.87–0.99)	0.03*	1.12 (1.06–1.18)	< 0.001***
Serum SAA levels	0.41 (0.27–0.62)	< 0.001***	0.82 (0.54–1.21)	0.319	1.72 (1.21–2.42)	0.002**	0.43 (0.27–0.69)	< 0.001***	0.88 (0.56–1.34)	0.548	1.49 (1.00–2.20)	0.048*

The covariates included age, glucose level, serum potassium level, WBC, and SAA (continuous), gender, history of hypertension, history of diabetes, smoking, and alcohol consumption (binary).

The odds ratio (OR) of SAA was logarithmically transformed as log(SAA + 1); each increase in standard deviation (SD) corresponded to this transformation.

The asterisk indicates statistical significance (the p-value is reported as median and interquartile range). ICH, intracerebral haemorrhage; NIHSS, National Institutes of Health Stroke Scale; GCS, Glasgow Coma Scale. ⁎ *p* < 0.05. ⁎⁎ *p* < 0.01. *** *p* < 0.001. OR, odds ratio; CI, confidence interval.

Patients who died during hospitalisation or those who were comatose on discharge exhibited higher SAA levels (9.5% vs. 5.2%); however, this difference was not statistically significant (*p* = 0.112) (Table 3). Taken together, the findings revealed that SAA levels were associated with poor functional outcomes in patients with ICH. Elevated SAA levels were associated with SBI following ICH. Figure 4A shows the representative CT imaging diagnoses of the patients included in this analysis.

**Table 3:** Prognostic analysis between the favourable prognosis (conscious at discharge) group and the poor prognosis group (in-hospital death or coma at discharge).

Serum SAA levels (mg/L)	Number	Result	Poor (*n* = 95)	*p*-value
		Favourable (*n* = 459)		
0–80	521	435 (94.8%)	86 (90.5%)	0.112
81–500	33	24 (5.2%)	9 (9.5%)	

Chi-square test, *p* > 0.05, no statistical significance.

### 3.3 Relationship between elevated SAA levels and SBI

After ICH, proinflammatory cytokines are involved in the process of SBI. Therefore, in this study, we monitored the dynamic changes in SAA levels in 19 patients with ICH within 24 h of admission and on days 3 and 7 after the first ICH. These 19 patients did not differ significantly from the remaining intracerebral haemorrhage patients in terms of age, sex, hypertension, diabetes, blood glucose, serum potassium, and SAA levels (all *p* > 0.05), indicating that this subsample is representative of the larger group (Supplementary Table S1). Using these data, we analysed the relationship between SAA levels at different time points and the severity of neurological deficits. SAA levels were significantly higher on days 3 and 7 after ICH than at admission (*p* < 0.001) (Figure 4C). These results suggest that day 3 may be a critical time window during which SAA exerts its pathophysiological effects.

## 4 Discussion

For the first time, our study dynamically monitored SAA levels in ICH patients during hospitalisation, including within 24 h of admission and between days 3 and 7. SAA levels significantly changed between admission and day 3 and between admission and day 7, but not between days 3 and 7. In this study, we also demonstrated that SAA levels were significantly elevated in ICH patients compared to healthy controls. Moreover, SAA levels were positively correlated with the NIHSS score and negatively correlated with the GCS score in ICH patients, indicating a relationship with neurological impairment. Our results are in line with the findings of Huangfu et al., who demonstrated that SAA levels are associated with a poor prognosis at 3 months after aneurysmal subarachnoid haemorrhage (12)_._ These findings confirm the important role of SAA levels in the development of SBI and have significant implications for the clinical treatment of ICH.

Increasing evidence suggests that inflammation plays an essential role in SBI development (17). Following ICH, red blood cells undergo degradation, releasing free haemoglobin. This process promotes oxidative stress, activates inflammatory cascades, and ultimately leads to neurological dysfunction (18). Proinflammatory cytokines [including IL-1, IL-6, TNF-*α*, interferon-*γ*, and transforming growth factor-*β* (TGF-β)] trigger the production of acute-phase proteins (A-SAA, SAA1.1, and SAA2.1) (19), the secretion of inflammasomes and the release of chemokines. These processes, in turn, activate leukocytes, triggering a cascade of inflammatory mediators. Zhu et al. reported that SAA1 is significantly upregulated in extracellular vesicles (EVs) associated with ICH. The use of anti-SAA1 monoclonal antibodies reduces the number of proinflammatory microglia and the infiltration of peripheral leukocytes, thereby alleviating cerebral oedema and improving neurological function in ICH mice (20). Interestingly, this study revealed that WBC counts were higher in the ICH group than in the control group, further supporting the idea that elevated SAA levels may exacerbate neuroinflammatory damage.

Previous studies have shown that SAA can stimulate the activation of the NOD-like receptor family pyrin domain-containing 3 (NLRP3) inflammasome and the production of IL-1 in microglia; this finding suggests that SAA may play a key role in the pathological effects of stroke in the context of systemic inflammation in the brain (19). Similarly, SAA has been detected in the brain tissue of patients with Alzheimer’s disease (AD). Inflammation promotes amyloid formation, and systemic inflammation increases cytokine expression in the brain; this triggers or exacerbates amyloid deposition by activating microglia and/or amyloid-associated proteins. Research has shown that inflammation plays a direct role in the process of amyloid deposition in the brain (21). One study confirmed that SAA1 significantly enhances Th17 cell differentiation in an experimental model of autoimmune encephalomyelitis, which leads to inflammatory disease (22). SAA may amplify the proinflammatory cascade associated with ICH by inducing the activation of the NLRP3 inflammasome and upregulating the expression levels of chemokines (CCL-3 and CCL-5) in microglia, thereby further exacerbating neurological deficits (19). These findings indicate the importance of SAA in acute brain injury and suggest a potential role for SAA in ICH.

Our study has several limitations. First, as a retrospective single-centre study, it represents only a subset of ICH patients at our hospital, which limits the generalisability of the findings. Second, as this was a retrospective observational study, we did not conduct a systematic stratified analysis of haemorrhage sites, such as the ventricles, basal ganglia, and frontal and temporal lobes, which may affect the reliability of the study. Furthermore, owing to the distribution characteristics of NIHSS scores in this study, performing a more appropriate ordinal logistic regression analysis was not possible. The findings of this study primarily reflect the clinical course of patients receiving non-surgical treatment. The use of SAA as a biomarker in surgical patients requires further investigation. Future prospective multicentre cohort studies should be conducted to validate and expand upon the findings of this study.

## 5 Conclusion

SAA levels are elevated in patients with ICH and are associated with prognosis. According to dynamic monitoring of levels, day 3 may be a critical time point for changes in SAA levels. These findings provide new insights into the inflammatory dynamics following ICH and indicate the optimal timing for anti-inflammatory treatment.

## Data Availability

The raw data supporting the conclusions of this article will be made available by the authors, without undue reservation.
