# Supplementary material for: Association between elevated serum amyloid a levels and clinical outcomes in intracerebral haemorrhage: a retrospective study
Source: Front Neurol. 2026 Jul 9;17:1806993. doi: 10.3389/fneur.2026.1806993 (PMC13391882; doi:10.3389/fneur.2026.1806993)
Supplement: Supplementary file 1 [file Supplementary_file_1.DOCX]

**Supplemental Material**

**Table S1.** The chart of this study is the baseline characteristics of the ICH dynamic monitoring group and the ICH non-dynamic monitoring group.

| **Characteristic** | **ICH dynamic monitoring group** (n = 19) | **ICH non-dynamic monitoring group** (n = 535) | ***p value*** |
| --- | --- | --- | --- |
| Male, n (%) | 16(84.2%) | 342(63.9%) | 0.069 |
| Age, years (mean ± SD) | 64.32 ± 12.21 | 63.62 ± 13.34 | 0.889 |
| Hypertension, n (%) | 14(73.7%) | 332(62.1%) | 0.304 |
| Diabetes mellitus, n (%) | 3(15.79%) | 54(10.09%) | 0.422 |
| Current smoking, n (%) | 5(26.3%) | 107(20%) | 0.501 |
| Alcohol consumption, n (%) | 4(21.1%) | 81(15.1%) | 0.482 |
| Plasma glucose level (mmol/L) | 9.47 ± 3.83 | 8.60 ± 3.10 | 0.370 |
| Plasma potassium level (mmol/L) | 3.66 ± 0.40 | 3.80 ± 0.49 | 0.118 |
| Blood leucocyte count (×109/L) | 7.68 ± 2.58 | 9.56 ± 4.23 | 0.055 |
| Serum SAA levels (mg/L) | 3.0 [1.7, 5.2] | 3.1 [1.2, 9.2] | 0.999 |

Notes: Age was reported as the mean ± standard deviation and qualitative data were presented as counts (proportions). Intergroup comparisons of various variables were conducted using the χ2 test for qualitative data, and Mann–Whitney U test for quantitative data. Non-normal distributions are characterized by the median [interquartile range]. Serum SAA levels, baseline level. no statistical significance (*p* > 0.05).

**Figure S1.**

**
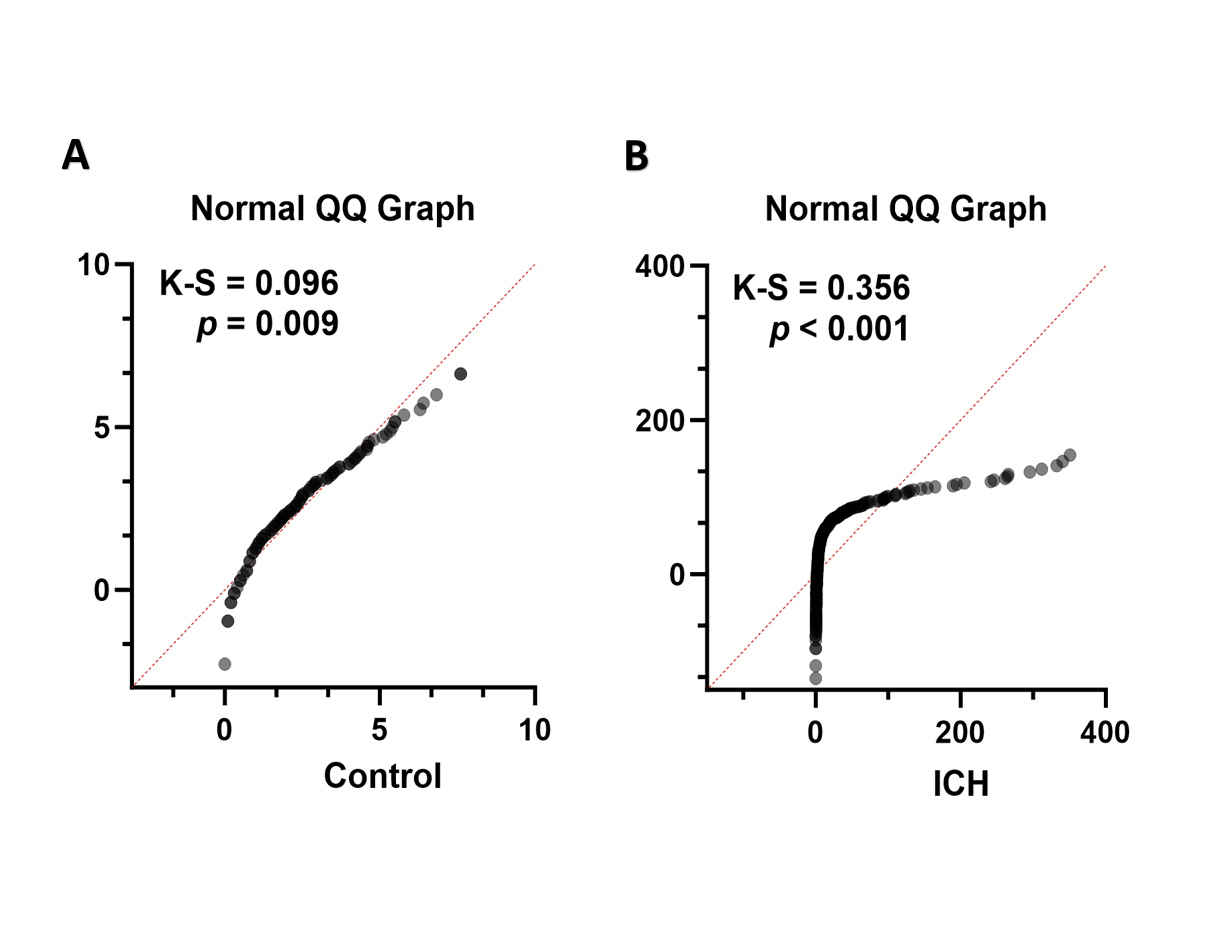
**

**Figure S1. QQ plot of the Kolmogorov–Smirnov normality test for serum SAA levels.** (A) Control group and (B) ICH group: to determine whether the data follow a normal distribution. ICH, intracerebral haemorrhage.

**Figure S2.**


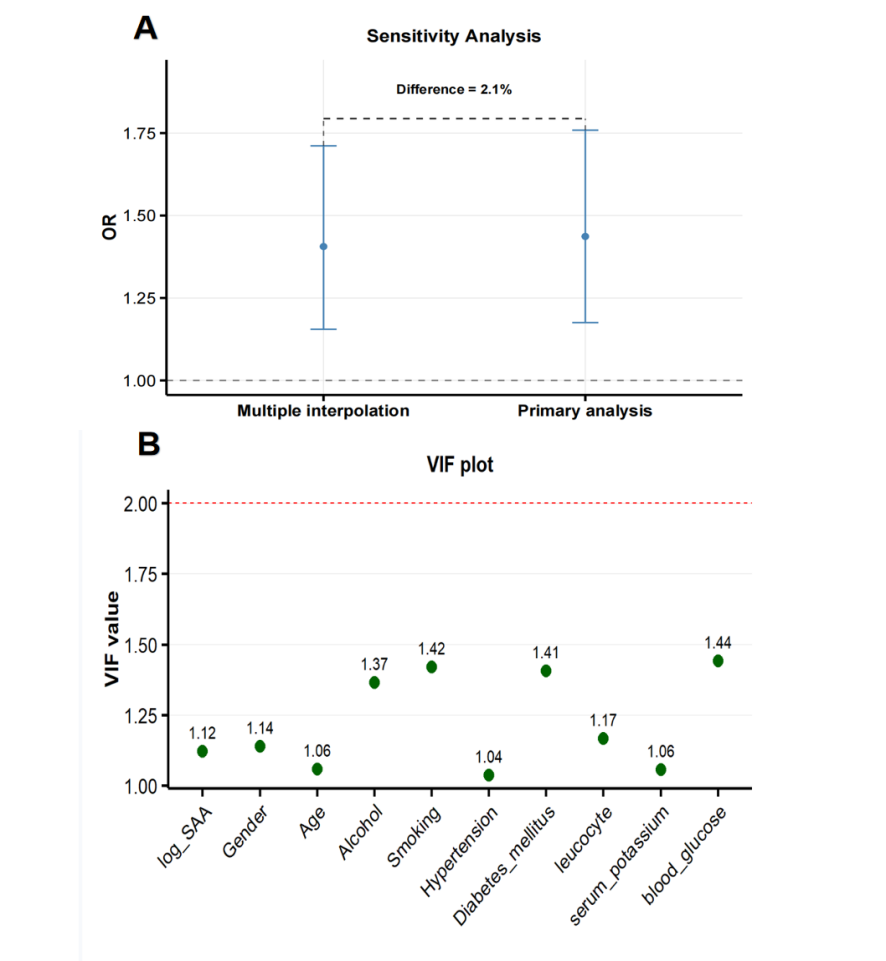


**Figure S2. Assessing the robustness of research findings.** (A) Multiple interpolation sensitivity analysis evaluates the impact of missing data on model stability. (B) Calculate VIF to assess multicollinearity among independent variables. VIF, variance inflation factor.
